# The Exciting Causes of Caries

**Published:** 1856-12

**Authors:** J. Taft


### THE EXCITING CAUSES OF CARIES.

BY J. TAFT.

(Coutinued from July number.)

When there is a predisposition to caries, any of the exciting causes will act with more vigor. Teeth that are well constituted, and have retained unimpaired health and vitality, often withstand the influence of injurious agents that would, in less favorable cases, destroy them in a very short time. The immediate cause of decay is the action of agents chemically upon the teeth.

We do not propose to enter into an examination of the behavior of the various agents that act chemically upon the teeth. That subject would open up a vast field for exploration—it yet remains unexplored—one that would far exceed the limits of the present paper.

There are several sources of these agents.

The vitiated secretions of the mouth, saliva and mucus. Abdominal secretion from the stomach. The decomposition of animal and vegetable substances in the mouth. Acids taken with food. Acids administered as medicines. Agents resulting from galvanic action.

Sometimes the secretions of the mouth are wholly of an acid character; then these natural products, thus vitiated, become instruments of mischief.

The natural condition of the *mucus* is acid, but of the saliva alkaline; so that any injurious influence the mucus alone might exert is counteracted by the saliva. But when the saliva and mucus are both acid, the teeth must suffer.

These secretions may become vitiated by the inability of the glands, on account of disease or an enfeebled condition, to perform their function perfectly. Or the blood may be in an imperfect condition, and the glands unable, on that account, though they were healthy,—which they seldom are in such cases—to elaborate healthy saliva.

When the fountain is corrupt, the stream can not be pure.

Thus any thing that will produce an abnormal condition of the blood, is paving the way for decay of the teeth.

An unhealthy condition of the blood frequently affects injuriously the secretive apparatus; and in this way a double mischief is accomplished.

To the pernicious influence of the saliva it may be objected, that all parts of the teeth would be similarly affected. But that it is otherwise is apparent, when we consider that the teeth are frequently not all equally well organized in the different parts; some points are not so well protected as others; and again, between the teeth, the saliva, with foreign substances, will be retained, and unite their influence upon the eeth.

In cases where there is a large amount of viscid saliva constantly flowing, the teeth decay very rapidly. The decay is of a light color; indeed, in many cases, it is difficult to distinguish it from the undecomposed dentine by its color.

The gastric fluid often becomes changed by irritation or disease of the stomach, so that the function of the latter is very imperfectly accomplished, and fermentation of the food occurs; and the production of agents that will act injuriously upon the teeth is the result.

In dyspeptics, these agents are often brought in contact with the teeth by eructations and vomiting.

The imperfect gastric fluid, which contains a large amount of hydrochloric acid, is also thus brought into contact with the teeth. It acts with great violence upon them. After food, commingled with this secretion, is ejected from the stomach, the teeth will be found eroded all over their surfaces.

Dyspeptics can appreciate the remark upon this subject.

If the teeth are not of superior organization in such cases, they will be destroyed in a short time. Their surfaces, thus roughened, afford a good lodgment for foreign substances upon all parts of them.

The agents that most commonly act injuriously upon the teeth are formed in the mouth, by the decomposition of animal and vegetable matter about the teeth. By this process, elements are eliminated, which form new combinations that operate as refined instruments in the destruction of the teeth.

Favorable conditions exist in the mouth for the decomposition and also for the formation of new cmpounds. There is heat and moisture, and each in such amount as to facilitate decomposition. Both of these, and particularly the former, facilitate the action of any acid upon the dentine.

The character of the saliva and mucus will very much modify the decomposition of foreign substances in the mouth. If they are both acid, the decomposition will be much more rapid, and the effect far greater.

Again, it is sometimes the case that the salivary glands are comparatively inactive, except when particularly excited to action, and yet the mucous glands still active, eliminating their secretion; and in this way the mouth takes on an acid condition, just because there is not saliva sufficient to neutralize the mucus. Decomposition of foreign substances would go on more rapidly in this condition than almost any other.

There are many cases in which there is a copious flow of saliva, and the decay very rapid; this is in consequence of the acid condition of both the secretions, or from rapid decomposition of foreign substances in the mouth.

With the food, doubtless, there are acids taken, that act directly upon the teeth. Acetic acid, or common vinegar, is used frequently by almost all persons, and by many in great quantities. It acts with great rapidity upon the teeth.

Prof. Westcot says: "Acetic and citric acids so corroded the enamel, in forty-eight hours, that much of it was easily removed with the finger-nail." He also remarks: "Malic acid, or the acid of apples, in its concentrated state, also acts promptly upon the teeth." Now, these acids are brought into frequent contact with the teeth, in the use of many kinds of food.

Sulphuric acid is often used in the manufacture of vinegar, so that we have the sulphuric alone, or in connection with the acetic. Sulphuric acid acts with greater energy upon the teeth than acetic. Acetic acid also facilitates the decomposition of food in the mouth. From this process this acid is an abundant product.

After eating apples which contain a great amount of malic acid, the teeth will be corroded all over their surfaces. These acids act on the enamel to some extent, and when it is very thin, though it may not all be removed from any particular point, yet its integrity is destroyed, so that it may soon be fractured, and permit injurious agents to come in contact with the dentine.

The dentine will yield far easier to the action of acids than the enamel. Points not well protected by the enamel, will be rapidly affected by acetic, malic, and sulphuric acids. These, in decayed cavities, will produce rapid results. These agents should be avoided as much as possible, and when used, the utmost care should be exercised, for the cleanliness of the teeth. It would be a safe course to use some neutralizing agent after the use of any acids with food.

During mastication, there is an increased amount of saliva, which, if it is in a healthy state, will tend to neutralize any acid that may at the time exist in the mouth; and also, by its flow, to remove foreign snbstances from the mouth.

Salts may become decomposed in the mouth; the acid then would act upon the teeth. They *will* become decomposed when the acid of the salt has a stronger affinity for any element of the tooth bone, than it has for the base with which it has been combined.

Many medical preparations contain agents peculiarly offensive to the teeth. A great number of these contain acids, and not in homeopathic dilutions either. The acids most commonly administered in medical preparations are hydrochloric, nitric, sulphuric, acetic, tartaric, and citric, any one of which will produce direct and rapid decomposition of the dentine, even without the aid of the temperature of the mouth; but this will very much facilitate their action.

These are often administered by physicians, without any regard to their nature, or their influence upon the teeth. Sometimes they are administered through a tube; this generally does not amount to any thing as a precautionary measure, for commonly the fluid passes through all parts of the mouth. Sometimes the mouth is rinsed with water; this amounts to a dilution, and not to an entire removal. To entirely counteract the injurious influence upon the teeth, an alkaline solution should be used after the administration of such medicines.

Galvanic action is a cause of decay of the teeth, only so far as it is the means of decomposing compounds that are in the mouth, and according to the laws of affinity, other combinations are formed. Some of these are highly injurious to the teeth.

The elements, hydrogen, nitrogen, and oxygen, may be set free from animal and vegetable substances; these at once seek other elements with which to combine. The nature of that combination will be governed by the elements, and by the accompanying circumstances.

These compounds will frequently be of an acid character. Such an arrangement may exist as will *keep up* galvanic action continually, and its legitimate effects be as constantly exhibited upon the teeth. This ceaseless process can not but make its mark.

A happy arrangement for galvanic action exists, when there are two or three kinds of metals in the mouth at the same time; and particularly those differing in their affinities for oxygen. Sometimes three or four kinds of metals are used in filling the teeth of the same mouth. Sometimes fillings of one metal, and a plate of another; and sometimes plates of so low caret are used, that they oxidize rapidly in the mouth, without the aid of any other metal.

These are some of the agents that produce caries of the teeth, and the sources from whence they originate. It is not maintained that in these remarks all the causes of decay have been referred to; but it is suggested that some of the most prominent have been pointed out. A close examination of even these, in all their details, has not been pressed. There are various subjects untouched here, connected with caries of the teeth, ample enough in their ramifications, each, for an extended essay.

